# Genome-wide genetic architecture for plant maturity and drought tolerance in diploid potatoes

**DOI:** 10.3389/fgene.2023.1306519

**Published:** 2024-01-31

**Authors:** Bourlaye Fofana, Braulio Soto-Cerda, Moshin Zaidi, David Main, Sherry Fillmore

**Affiliations:** ^1^ Charlottetown Research and Development Centre, Agriculture and Agri-Food Canada, Charlottetown, PE, Canada; ^2^ Departamento de Ciencias Agropecuarias y Acuícolas, Universidad Católica de Temuco, Temuco, Chile; ^3^ Núcleo de Investigación en Producción Alimentaria, Facultad de Recursos Naturales, Universidad Católica de Temuco, Temuco, Chile; ^4^ Kentville Research and Development Centre, Agriculture and Agri-Food Canada, Kentville, NS, Canada

**Keywords:** diploid potato, climate change, drought, maturity, GWAS, candidate genes

## Abstract

Cultivated potato (*Solanum tuberosum*) is known to be highly susceptible to drought. With climate change and its frequent episodes of drought, potato growers will face increased challenges to achieving their yield goals. Currently, a high proportion of untapped potato germplasm remains within the diploid potato relatives, and the genetic architecture of the drought tolerance and maturity traits of diploid potatoes is still unknown. As such, a panel of 384 ethyl methanesulfonate-mutagenized diploid potato clones were evaluated for drought tolerance and plant maturity under field conditions. Genome-wide association studies (GWAS) were conducted to dissect the genetic architecture of the traits. The results obtained from the genetic structure analysis of the panel showed five main groups and seven subgroups. Using the Genome Association and Prediction Integrated Tool–mixed linear model GWAS statistical model, 34 and 17 significant quantitative trait nucleotides (QTNs) were found associated with maturity and drought traits, respectively. Chromosome 5 carried most of the QTNs, some of which were also detected by using the restricted two-stage multi-locus multi-allele-GWAS haploblock-based model, and two QTNs were found to be pleiotropic for both maturity and drought traits. Using the non-parametric *U*-test, one and three QTNs, with 5.13%–7.4% phenotypic variations explained, showed favorable allelic effects that increase the maturity and drought trait values. The quantitaive trait loci (QTLs)/QTNs associated with maturity and drought trait were found co-located in narrow (0.5–1 kb) genomic regions with 56 candidate genes playing roles in plant development and senescence and in abiotic stress responses. A total of 127 potato clones were found to be late maturing and tolerant to drought, while nine were early to moderate–late maturing and tolerant to drought. Taken together, the data show that the studied germplasm panel and the identified candidate genes are prime genetic resources for breeders and biologists in conventional breeding and targeted gene editing as climate adaptation tools.

## 1 Introduction

Climate is changing and growing seasons are predicted to become hotter and drier, and crop productivity will be drastically reduced (Dietz et al., 2021; Hill et al., 2021). Cultivated potato (*Solanum tuberosum*) has a shallow root system and is well known to be highly susceptible to drought (Gervais et al., 2021; Nasir and Toth, 2022). The driving forces that determine the direction of water movement from the soil to the plant, throughout the plant, and from the plant to the atmosphere are the water potential gradients (Xiong and Nadal, 2020; Dietz et al., 2021). When the water potential of the soil is more negative than that of the roots as a result of water shortage, the root loses water to the soil, shoot, and atmosphere, leading to loss of cell turgidity and plant tissues wilt, following a decrease in water potential in the entire plant (Martinez-Vilalta et al., 2017). Water stress affects all phenological stages of plant growth and development, affecting vegetative and generative growth and yield (Dietz et al., 2021), and its effects are correlated with its intensity, duration, and timing for each crop (Patrick and Stoddard, 2010; Waser and Price, 2016; Hu et al., 2019; Aliche et al., 2020; Dietz et al., 2021).

Modern potato cultivars are known to be extremely susceptible to drought, and the potato crop yields are anticipated to be drastically impacted in many areas of the world under the predicted hotter and drier changing climate (Dietz et al., 2021; Hill et al., 2021). Indeed, although cultivated potato has a higher water use efficiency (105 L/kg potato produced) compared to many other major crops such as corn (710 L/kg), rice (1048 L/kg), and wheat (1159 L/kg) (Nasir and Toth, 2022), its shallow root system (Van Loon, 1981) prevents water absorption from deeper soil layers during water shortage. Hence, root length and volume, plant stem-type canopy, and leaf-type canopy characteristics have been reported as key water stress coping traits (Lahlou and Ledent, 2005; Schittenhelm et al., 2006; Aliche et al., 2018). Nonetheless, up until recently, breeding for drought resistance had not been a hot spot breeding objective *per se*, and potato varieties that are resistant to drought are currently limited (Sprenger et al., 2016; Martinez et al., 2021). Thus, with climate change and its predicted frequent episodes of high temperature (Dietz et, al., 2021) and drought (Sreeparvathy and Srinivas, 2022), potato growers will face increased challenges to achieving their predicted yield goals if new climate-adapted potato cultivars are not developed.

Currently, a high proportion of untapped potato germplasm remains within the diploid potato relatives, not yet fully explored for their abiotic stress resistance potentials. While, drought resistance genes have been reported in other crops (Shinozaki and Yamaguchi, 2007; Joshi et al., 2016; Soto-Cerda et al., 2020), to our knowledge, only very few studies have been reported on mapping genes conferring drought tolerance in potatoes (Anithakumari et al., 2012; Khan et al., 2015; Schumacher et al., 2021), all focused on bi-parental populations. Of the previous studies, only Schumacher et al. (2021) performed in controlled and field settings and, using an indirect yield trait (the deviation of the relative starch yield median—DRYM) in cultivated tetraploid potato, identified candidate genes located in very large (1.3 Mb–36.76 Mb) genomic regions associated with the DRYM drought-tolerance trait. To date, no candidate genes associated with drought tolerance or maturity, two key traits for potato crop adaptation to changing climate, have been identified in diploid potatoes.

Based on the current knowledge, cultivated tetraploid potato landraces belonging to *S. tuberosum*, group Andigena, have been shown to have tolerance to drought (Nasir and Toth, 2022). However, their use in breeding programs has been limited. It is also known that wild diploid potato relatives to cultivated tetraploid potatoes have co-evolved in their natural habitats while coping with biotic and abiotic stress (Berdugo-Cely et al., 2017; Bashir et al., 2021; Tiwari et al., 2022). We have previously developed an ethyl methanesulfonate (EMS)-mutagenized diploid potato germplasm collection derived from seven crosses involving one female and seven pollen donors in bi-parental crosses, resulting in a higher genetic diversity and novel phenotypic variants (Somalraju et al., 2018; Somalraju et al., 2020).

Recent breakthrough with next-generation sequencing technologies, genomics, and bioinformatics tools ha**s** led to a molecular marker, namely, single-nucleotide polymorphisms (SNPs), that has become popular in marker-assisted breeding in crops (Moose and Mumm, 2008; Kumar et al., 2012; Chung et al., 2017). Genotyping by sequencing (GBS) and whole-genome sequencing have become the common ways for SNP detection (Poland et al., 2012; He et al., 2014). Although whole-genome sequencing ensures higher genome coverage (Wang et al., 2020), it can be expensive for a large number of samples despite the drastic reduction in the sequencing cost (Schwarze et al., 2020). Since most applications do not require sequencing of each base in the genome (Pootakham, 2023), reduced-genome representation GBS (Elshire et al., 2011) has been proven as an efficient method for SNP genotyping (Poland et al., 2012; He et al., 2014; Berthouly-Salazar et al., 2016; D’Agostino et al., 2018; Favre et al., 2021), albeit some associated technical limitations such as read depth variations (Beissinger et al., 2013; Wang et al., 2020). Because of their genome-wide distribution, ubiquitous nature, abundance, reliability, and low cost (Rafalski, 2002; Ganal et al., 2009; Mammadov et al., 2012; Goodwin et al., 2016)**,** SNPs are now the most widespread genetic markers used in genome-wide association studies (GWAS) for QTL/quantitative trait nucleotide (QTN) mapping and candidate gene identification in the diverse germplasm panel of crops (Buntjer et al., 2005; Tibbs Cortes et al., 2021; You et al., 2022). Currently, to our knowledge, no study has been reported on the genetic architecture of the drought-tolerance and maturity traits of diploid potatoes in a field condition. We hypothesize that the EMS-mutagenized diploid panel will show a genomic architecture associated with drought-tolerance and maturity traits.

The objectives of this study were 1) to increase our knowledge of the genetic architecture of drought tolerance and maturity in a germplasm panel of EMS-mutagenized diploid potatoes grown under field conditions and 2) to identify candidate genes associated with each of the two traits. Using two GWAS statistical models and a non-parametric test, QTNs/QTLs associated with the traits were identified, and we showed that two SNP loci (Schr05_3842784 and Schr05_4617251) located on chromosome 5 are pleiotropic for maturity and drought. A total of 38 and 18 candidate genes were found co-located with the QTLs/QTNs associated with the maturity and drought traits, respectively. Most of these genes (53% and 50%) were located at a close (0.5–1 kb) distance to the significant SNP. The data show that the studied germplasm panel and the identified genes are prime genetic resources for breeders and biologists in conventional breeding and targeted gene editing in the context of climate change.

## 2 Materials and methods

### 2.1 Plant materials

The plant material consisted a 384 diploid potato germplasm panel including 47 wild types refreed to as control (CTL) and 337 EMS-mutagenized (EMS) clones (Supplementary Table S1) selected from an 814 germplasm collection, each corresponding to a clone, and previously developed and described by our group (Somalraju et al., 2018). The original 814 germplasm collection (Somalraju et al., 2018) has consistently been propagated in field each year since 2014 as a 5–6-hill plot without replication and phenotypic data collected for traits including germination, plant characteristics, maturity, and sensitivity to drought. Since 2017 (except 2020; due to the COVID-19 pandemic), a manageable size of clones (35–100 clones) have gone through a commercial-scale yield trial as a 25-hill plot each year, with three replications per clone/year and phenotyped, and tubers were graded following the Canadian Food Inspection Agency (CFIA) commercial standard. Antinutritional factors including glycoalkaloid and asparagine contents were also determined in a subset of the collection (Somalraju et al., 2020). All plants were grown under conventional agronomic practices at the Agriculture and Agri-Food Canada, Harrington Farm (PE, Canada). In 2021, the core 384 germplasm panel was formally established and tested in a separate trial for scab resistance as a 5–6-hill small plot without replication. The clones in the panel were selected in such a way that selections from a particular bi-parental cross were not overrepresented, both for the wild types and EMS-mutagenized clones (Table 1; Supplementary Table S1) and based on prior phenotypic data (unpublished), using a combination of tuber traits such as yield potential, tuber appearance, and antinutritional factors including the glycoalkaloid and asparagine contents.

**TABLE 1:** Frequency distribution of diploid potato clones used in the study.

Type	Cross #	Number of clones	Proportion of clones (%)^a^
48 CTL	1	6	12.5
	2	8	16.7
	3	5	10.4
	4	6	12.5
	5	7	14.6
	6	9	18.8
	7	7	14.5
336 EMS	1	43	12.8
	2	61	18.0
	3	40	12.0
	4	37	11.0
	5	48	14.3
	6	44	13.1
	7	63	18.8

^a^ Proportion determined within each type.

In general, the 384 clones in the panel were planted in at least one to three trial sites each year since 2021, and phenotypic data were collected. For each trial site per year, five to six plants per clone were planted. The plot length varied from 1.5 m (for 814 clones in the germplasm collection and 384 core panel) to 7.5 m (yield trial), with the spacing between plants in each plot and between plots of 25 cm and 1 m, respectively. Plots were arranged in a complete randomized design with one replication in the germplasm collection and 384 core panel and three replications in the yield trial. For each trial, fertility (100 kg of 15-15-15-2 NPK and Mg) and pesticide (Admire, Capture/Cimegra, Bravo/Orondis/Reason, Superior oil) treatments were applied as performed following the standard procedures as for conventional commercial potato production. No irrigation was performed in the experiment, and climate data were recorded for 2020–2022 growth seasons (Supplementary Figure S1). For each trait evaluated in the study, data were collected on the 384 clones at least from one to three trial sites.

### 2.2 Maturity and drought-tolerance trait phenotyping

From 2016 to 2022, the germplasm collection was planted each year between May 25th and June 15th, and the plant emergence and senescence dates were recorded. Plant maturity data were systematically recorded and rated for all the 384 clones in years 2021 and 2022, at different time points during the growing season in mid-August, late August, and mid to late September. Year 2020 was an extreme dry growth season (Supplementary Figure S1), with severe and long drought episodes during all plant phenological stages (21 days from June 11 to July 1, 0 mm of average precipitation and 18.2°C average temperature; 16 days from July 12 to July 27, 0.23 mm of average precipitation and 19.6°C; and 25 days from July 31 to August 24, 0.07 mm of average precipitation and 20.64°C). Year 2021 was considered a normal growing season with normal rain fall throughout the growing season, whereas 2022 was considered a mild dry season, with two short drought episodes of 8 days each (July 20 to July 28 and July 31 to August 7) at tuber initiation and bulking stages and a long episode of 19 days later in the season (August 28 to September 15).

Plant maturity was systematically monitored for each clone grown at 1–3 sites during the 2021 normal and the 2022 fairly normal growing seasons. Clones were rated 1 (early) when 60%–80% of plants per plot have senesced or are senescing, 2 as moderate to late when 40%–60% of plants per plot have senesced or are senescing, and 3 (late) when less than 40% of plants per plot have senesced or are senescing.

Drought phenotypic data were collected only in years 2020 and 2022 after the drought episodes (Supplementary Table S2). Plants were rated after 8–25 days of drought episodes over the 2 years. In 2020, plant rating was performed after 21 (June 11–July 1; 0 mm average precipitation and 18.2°C average temperature), 16 (July 12–July 27; 0.23 mm average precipitation and 19.6°C average temperature), and 25 (July 25–August 25; 0.07 mm average precipitation and 20.64°C average temperature) days of drought episodes. In 2022, plant rating was performed after 8 (July 20–July 28; 0.04 mm of average precipitation and 22.3°C average temperature), 8 (July 31–August 7; 0.41 mm of average precipitation and 22.05°C average temperature), and 19 (August 28–September 15; 0.24 mm of average precipitation and 17.8°C average temperature) days of drought episodes. Plants were rated as 1 for susceptibility when 60%–80% of the plants per plot have dried or wilted and as 2 for tolerance when less than 10% of the plants per plot have wilted.

Statistical analyses were performed on the maturity and drought stress data collected from the 384 clones over 2 years (2021 and 2022) in 1–3 trial sites. Descriptive statistical analyses were performed in GeneStat (version 12.1 for Windows) to test differences between factors clones and years, using a mixed model, with no random effects and fixed effects of clone and year, focusing on clone differences as main effects. The significance for fixed terms was evaluated using a Wald statistic test (*p* < 0.05). Thereafter, the data were log-transformed for normalization as required in *R*, version 4.2.0, and the means and best linear unbiased predictors (BLUPs) of the lines for each trait were calculated for the 2 years using *R* library “Phenotype” version 0.1.0 (https://rdrr.io/cran/Phenotype/man/blup.html; Piepho et al., 2008; Zhao, 2020). Whenever possible, phenotype diagnostics through distribution plots were generated using Genome Association and Prediction Integrated Tool (GAPIT) version 2 (Tang et al., 2016). A principal component analysis was performed for population structure and kinship, and the covariates were used for QTL/QTN mapping using the Genome Association and Prediction Integrated Tool–mixed linear model (GAPIT–MLM) GWAS, version 3 (Yu et al., 2006; Lipka et al., 2012; Tang et al., 2016; Wang and Zhang, 2021) and the restricted two-stage multi-locus multi-allele genome-wide association study (RTM-GWAS) statistical models (He et al., 2017). Means and BLUP values were used for GWAS analyses.

### 2.3 Genotyping

#### 2.3.1 DNA extraction

Young leaf tissue (50–75 mg) samples were collected from each clone, immediately frozen in liquid nitrogen, and lyophilized in a freeze-dryer (for 48–72 h). Genomic DNA was extracted using a Qiagen DNeasy 96 Plant Kit (Qiagen, Germantown, MD, United States), following the manufacturer’s instructions. DNA quality was assessed on an agarose gel, and then, the DNA was quantified by fluorometry using the Quant-iT™ PicoGreen™ dsDNA Assay Kit (Thermo Fisher, Waltham, Massachusetts, United States) as recommended by the supplier and finally normalized to 5ng/uL. As DNA quality and quantity varied widely, and for some samples it was not possible to achieve a concentration of 5ng/uL, these samples were included with minimal dilution in an attempt to derive as many sequencing reads as possible. All samples were used for library construction.

#### 2.3.2 Genotyping by sequencing

GBS reduced-representation sequencing libraries were constructed following Elshire et al.’s (2011) protocol by Platform Genetics (Vineland Station, ON, Canada). In brief, three restriction enzymes (*Pst*I, *Nsi*I, and *ApeK*I) were tested, and adaptor concentrations were empirically titrated. *Pst*I was chosen, and DNA digestion was performed with *Pst*I. The ligation was performed using 0.6 ng/uL adaptors that produced an acceptable size distribution. One GBS library was constructed for each of the 384 clones, and a total of 384 GBS libraries were produced. These 384 libraries were arranged in batches of four full 96-well plates, and samples from each plate were subsequently pooled in equimolar ratios for a total of four pooled libraries. Each of these four pooled libraries were further diluted and normalized to 30 nM, and 20 µL from each normalized library was pooled to form a final pooled library that was sequenced as paired-end (PE150) through the 240 Gb SP flow cell by Novogene (Genome Quebec, Montreal, QC, Canada) using the Illumina NovaSeq 6,000 sequencing platform (Illumina, San Diego, CA). Only the forward reads were used for downstream GBS analyses. The raw data can be found under SRA accession PRJNA1032882 in the NCBI database.

#### 2.3.3 SNP variant data analyses

The raw FASTQ read files were de-multiplexed into multiple files by barcode, and a custom Perl script was used to extract usable reads from each BAM file. The reads were then aligned and mapped against the potato (*S. tuberosum*) reference genome (http://spuddb.uga.edu/dm_v6_1_download.shtml) (Pham et al., 2020) using the Burrows–Wheeler Alignment (BWA), BWA MEM algorithm version 0.7.13r1126 (Li and Durbin, 2010; Li, 2013). SNP marker variants were identified using the GATK SNP calling pipeline (Van der Auwera and O’Connor, 2020; https://software.broadinstitute.org/gatk/) and the high contiguity of the potato reference genome (http://spuddb.uga.edu/dm_v6_1_download.shtml) for calling SNPs. A GQ > 20 and read depth >5 were set as the threshold to retain a genotype call. SNPs with call rate <50%, SNPs for which >95% of the genotype calls were identical, and SNPs where a minor allele frequency (MAF) is <5% were filtered out. This ‘cleaned high-quality dataset’ was used to estimate the decay of linkage disequilibrium (LD), within a sliding window size distance of 10,000 kb for the average LD plot and the whole chromosome as a sliding window size for LD by chromosome (https://www.cog-genomics.org/plink/1.9/ld), and the SNP coordinates were converted to the chromosome scale of the potato reference genome using GATK (Van der Auwera and OConnor, 2020). Using Plink 1.9 (Chang et al., 2015; (https://www.cog-genomics.org/plink/1.9/ld), the cleaned dataset was further pruned to produce uncorrelated ‘pruned markers’ so that no pair of markers on the same chromosome had a square correlation coefficient *r* ^2^ > 0.2.

#### 2.3.4 Population genetic structure and genome-wide association mapping

The genetic structure among the 384 accessions was computed using cleaned SNPs distributed across the 12 potato chromosomes using the GAPIT in *R* (Yu et al., 2006; Lipka et al., 2012; Tang et al., 2016; Wang and Zhang, 2021), TASSEL v 5.2.31 (Bradbury et al., 2007), and phangorn version 2.5.5 (Schliep, 2011). Using the cleaned SNPS, principal component (PC) and kinship analyses were performed in TASSEL v 5.2.31 (Bradbury et al., 2007), and a neighbor-joining (NJ) phylogenetic tree was built as previously described (Felsenstein, 1981; Felsenstein, 1985; Saitou and Nei, 1987) based on the uncorrelated pruned SNPs using phangorn, version 2.5.5 (Schliep, 2011). The number of subgroups was determined and presented as 2D and 3D PCs and kinship heatmap using the GAPIT (Lipka et al., 2012; Tang et al., 2016).

Genome-wide trait-to-genotype association mapping was conducted by using the GAPIT–mixed linear model (GAPIT-MLM) of GAPIT version 3 (Yu and Buckler, 2006; Lipka et al., 2012; Tang et al., 2016; Wang and Zhang, 2021) and the haplotype block-based RTM-GWAS (He et al., 2017) statistical models. The “cleaned” SNP dataset and three independent trait datasets (trait means, trait BLUPs, and traits separated by years) were used as inputs for association mapping using the GAPIT–MLM (Lipka et al., 2012) algorithm, with three principal components, as indicated by Scree plots drawn in the GAPIT *R* package. A significant trait-to-marker association threshold was set as the false discover rate (FDR)/Benjamini–Hochberg (B&H)-adjusted *p*-value < 0.05. GWAS outcomes were summarized and displayed using Manhattan plots, and the ability of GWAS statistical models to assess accuracy and to minimize false positive associations was tested with quantile–quantile (Q–Q) plots using the GAPIT as described and reported by Garcia et al. (2019). Using the GAPIT–MLM, for each individual and for each trait, the number of alleles at QTL, which increase the trait value, was counted, and the correlation between the number of trait-increasing alleles and trait value was determined. The correlation between the proportion of alleles at QTL (pQTN) that are trait-increasing and the trait value was also determined, excluding the missing genotype calls from the calculation.

RTM-GWAS QTL/QTN mapping was also performed using the three independent trait datasets (trait means, trait BLUPs, and traits separate), with each analysis reporting significant SNP loci at FDR <0.05. Manhattan plots and significant QTLs/QTNs were generated at the FDR adjusted *p*-value <0.05. Similar to the pQTN analysis using the GAPIT–MLM, the RTM-GWAS model was also used for pQTN analysis. Here, for each individual and each trait, the number of genotypes at QTL that increase or decrease the trait value was determined. The correlation between the number of trait-increasing genotypes and trait value and the correlation between the proportion of genotypes at QTL (pQTN) that are trait-increasing and trait value were determined, excluding missing genotype calls from the calculation. Significant QTNs obtained from the GAPIT–MLM GWAS analyses were further tested and retained if the mean for the two alleles (QTN effect) significantly affected the trait in all accessions. Mann–Whitney non-parametric *U*-tests (*p* < 0.05) were performed to remove the potential false positive QTNs using BlueSky Statistics (https://www.blueskystatistics.com/).

### 2.4 Candidate gene identification

Candidate genes co-located within a window of 500 bp–10000 bp on either side of the significant QTNs were scanned as previously described (You et al., 2022) and annotated based on the most recent release of the potato reference genome (Pham et al., 2020).

## 3 Results

### 3.1 Trait phenotypic distribution

**FIGURE 1:**
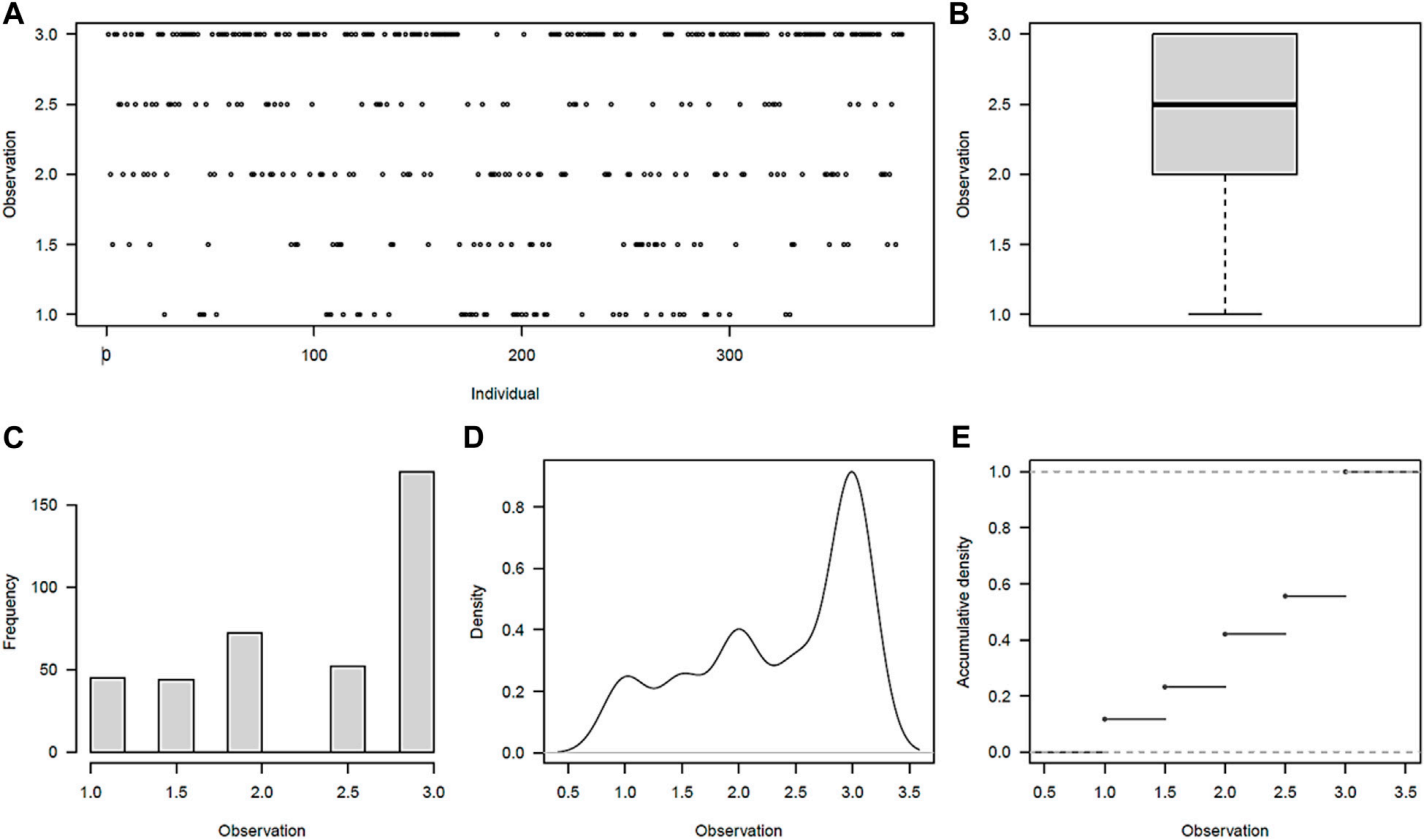
Maturity phenotype distribution among the 384 germplasm panel. **(A)** Visual distribution of individuals in maturity rating classes, **(B)** scatter plot of average distribution, **(C)** frequency distribution of individuals across maturity classes, **(D)** density plot of individuals across maturity classes, and **(E)** accumulation density distribution across maturity classes.

Maturity phenotype distribution was found to be skewed toward late maturity, with 44% (171) of the clones rating as late maturing (3.0 ± 0) (Figures 1A–E). The early (mean of 1.0 ± 0) and moderate-to-late (1.5 ± 0.70–2.5 ± 0.70) maturing potato clones accounted for 12% (45) and 44% (168), respectively. The observed grand mean was 2.34 ± 0.02. While 70% (268/384) of the clones showed no variations over the 2 years in the early- or late-maturity classes, a significant variation (*p* < 0.001) was observed for 30% of the clones in the moderate-to-late-maturity class. Overall, significant differences (*p* < 0.001) were observed between clones and between years for the traits (Supplementary Table S3).

**FIGURE 2:**
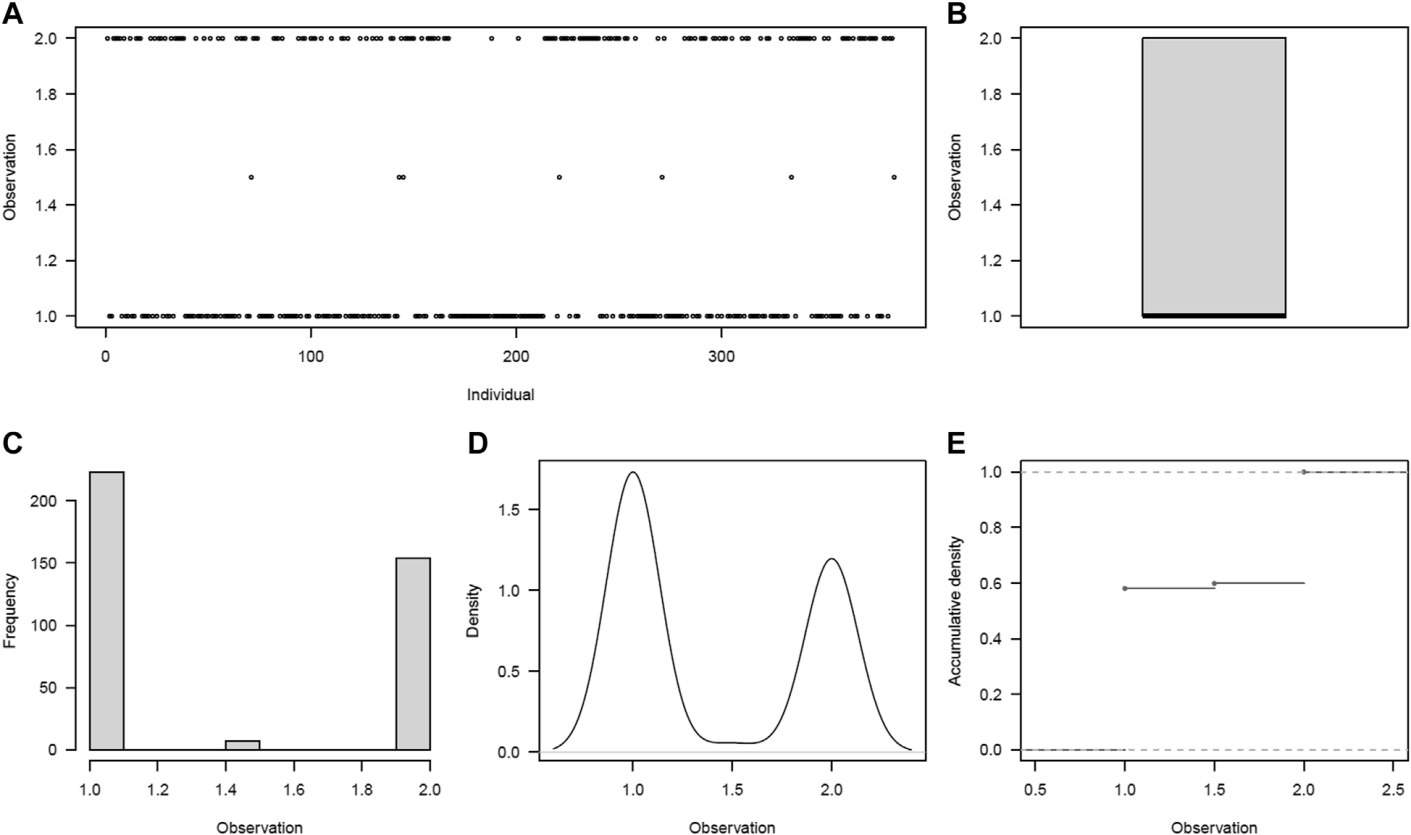
Drought phenotype distribution among the 384 germplasm panel. **(A)** Visual distribution of individuals in drought rating classes, **(B)** scatter plot of average distribution, **(C)** frequency distribution of individuals across drought ratings, **(D)** density plot of individuals across drought ratings, and **(E)** accumulation density distribution across drought ratings.

The drought trait, scored as a binary trait, showed a bimodal distribution (Figures 2A–E). Using both the mean and BLUP values, 58% (223/384) of the potato clones were susceptible (mean of 1.0 ± 0) to drought, while 2% (7 clones) and 40% (154 clones) appeared to be moderate (mean of 1.5 ± 0.70) to tolerant (mean of 2.0 ± 0), respectively. Rating in 98% of the clones was very consistent over the 2 years, with no significant difference (*p* = 0.06) observed between the 2 years. Significant differences (*p* < 0.001) were observed between potato clones for the drought trait (Supplementary Table S3). Overall, nine clones were rated as being early-to-moderate maturing and tolerant to drought, whereas 127 clones were found to be late maturing and tolerant to drought.

### 3.2 SNP discovery and the genetic structure of the population

**FIGURE 3:**
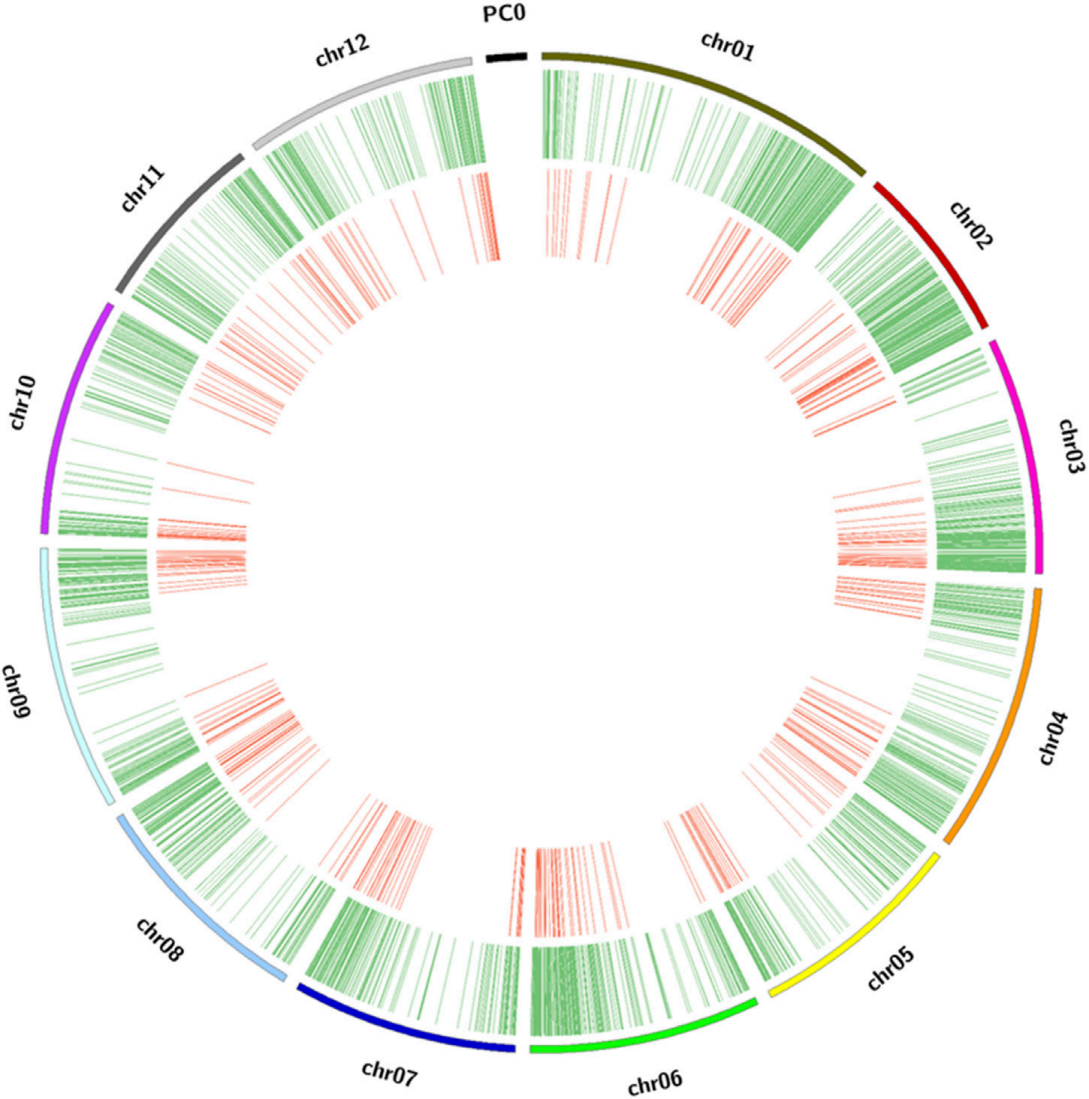
Distribution of SNP markers in the *Solanum tuberosum* genome. The outer ring of the diagram represents the chromosomes of *Solanum tuberosum*. The middle green ring shows the location of the cleaned SNPs on each chromosome, used for LD analysis. The inner red ring illustrates the chromosomal location of the pruned markers used for phylogenetic tree construction.

**FIGURE 4:**
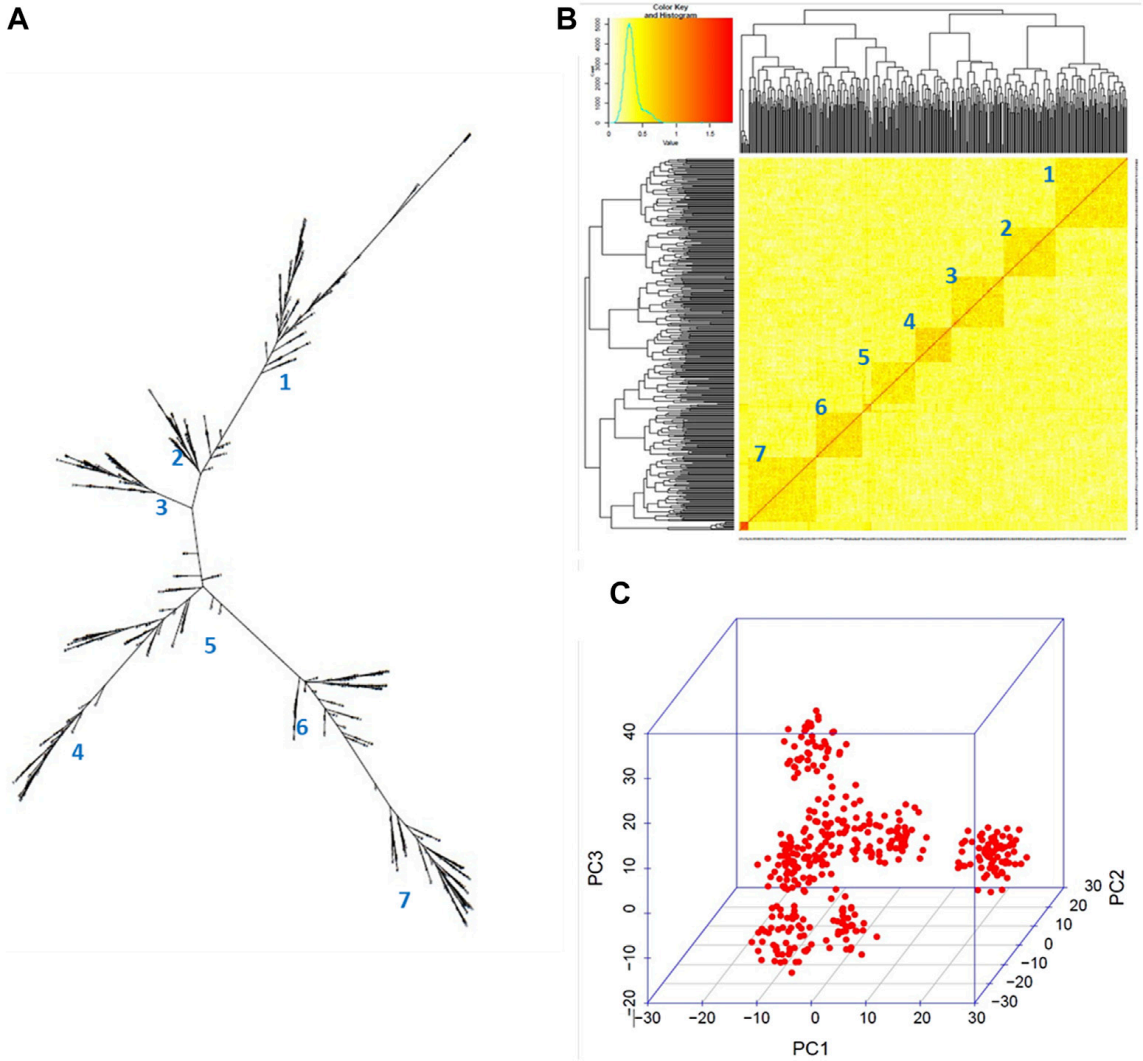
Genetic structure within the 384 germplasm panel. **(A)** Neighbor-joining phylogenetic tree, **(B)** kinship heatmap, and **(C)** 3D representation of the relationships between the clones depicted using the GAPIT in *R*. Seven subgroups can be observed.

The GBS sequencing produced clean mapped reads ranging from 3,635 to 2,703,393, with an average of 1,310,930 reads and an average depth coverage of 0.27X (range of 0.00074X–0.55X) among the 384 potato clones on the portion of the genome sampled through GBS. By aligning the clean reads to the reference genome, a total of 12,075 cleaned SNPs were detected. These SNPs were found well distributed across all 12 chromosomes of the potato genome, with an average of 1,006 SNPs per chromosome. A higher density was found on chromosome 1 (1,378 SNPs) and a lower density (722 SNPs) on chromosome 7 (Figure 3; Supplementary Figure S2). Here, the GBS depth coverage generated enough SNPs for an efficient genotyping as previously reported (Pootakham, 2023). After removing the monomorphic SNPs and those with segregation distortion >0.05, 11,605 SNPs were used for GWAS through the GAPIT–MLM GWAS and RTM-GWAS. These cleaned SNPs were further pruned to 455 SNPs (Figure 3) and used for phylogenetic tree construction, principal component, and kinship analyses, and the results showed five main groups and seven subgroups in the population (Figures 4A–C).

### 3.3 GWAS identification of QTL/QTN associated with maturity and drought traits

#### 3.3.1 Maturity

**FIGURE 5:**
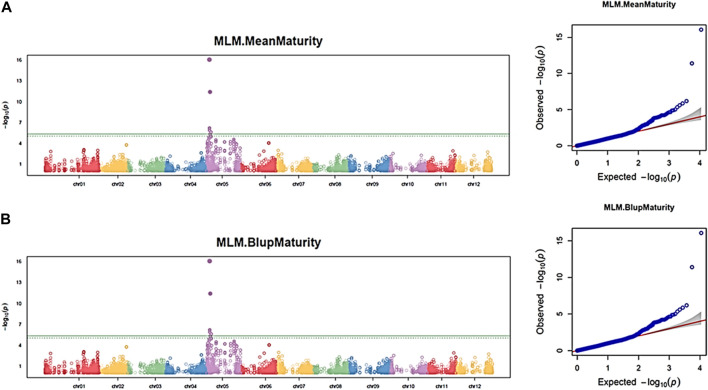
Manhattan and Q–Q plots showing QTN and chromosomal regions associated with maturity trait using the GAPIT–MLM model. Each panel corresponds to one dataset. **(A)** Maturity mean dataset; **(B)** maturity BLUP dataset. The green line indicates the FDR threshold cut off <0.05, and the solid green line indicates the Bonferroni threshold cutoff at 0.05.

**FIGURE 6:**
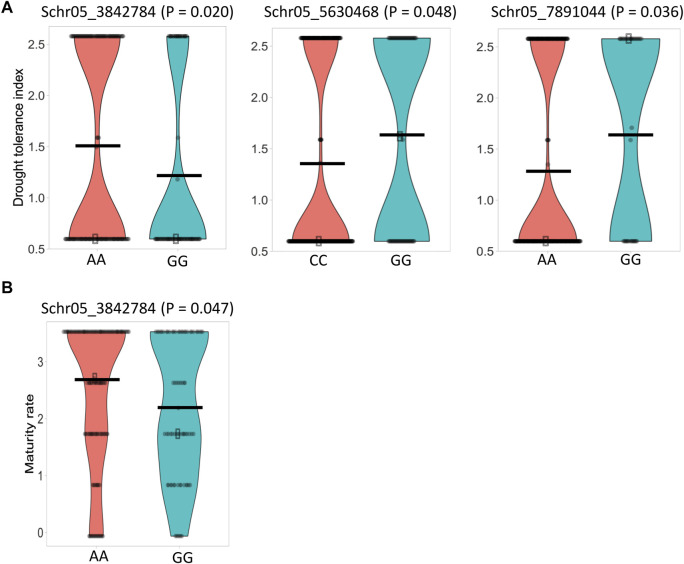
Violin plots illustrating the phenotypic differences between potato genotypes carrying different alleles of the significant SNPs. **(A)** Drought tolerance. **(B)** Plant maturity. Horizontal black bars represent the mean for each SNP allele. Statistical differences between alleles were tested using the Mann–Whitney non-parametric *U*-test (*p* < 0.05).

Using the 11,605 SNPs and the three independent datasets of the maturity trait, 34 significant SNPs (FDR <0.05) were associated with the trait on chromosomes 5 and 6 in each dataset. The Q–Q plot indicates a fairly well-fit GWAS model and minimal artifact bias from -log_10_ *P*) values >2.5 (Figure 5). Chromosome 5 carried 33 significant SNPs, and chromosome 6 carried one (Supplementary Table S4; Figure 5). The mapped significant SNPs showed large effects on maturity, varying from −0.75 to 0.87. Mann–Whitney non-parametric *U*-tests were conducted on the 34 significant SNPs. As expected, markers with higher *p*-values (>0.05) did not show significant allelic effects, while SNP Schr05_3842784 (*p* = 2.3e-05) that had 5.41% of phenotypic variation explained (PVE) exhibited significant positive effects for accessions harboring the AA allele (*p* = 0.047), displaying an SNP effect of 0.53 in increasing maturity rating, and consequently was retained for further analysis (Supplementary Table S4a; Figure 6B). Moreover, the SNP Schr05_4617251, although not found significant from the non-parametric *U*-test, showed 42% of PVE on maturity (Supplementary Table S4a).

Similarly, by performing a haploblock-based RTM-GWAS analysis using the same SNPs and phenotypic datasets, a total of 13 QTNs and 17 haploblock QTL loci were detected (Supplementary Table S4). Contrary to the GAPIT–MLM GWAS, the 30 QTNs/QTLs found in the RTM-GWAS model were distributed on 11 of the 12 potato chromosomes but not chromosome 4. Of these 30 QTNs/QTLs, nine (Block_chr05_4616945_4617251, chr05_11334011, chr01_9365386, chr03_47482310, Block_chr06_47240108_47240257, chr03_54033577, Block_chr07_10830500_10830536, Block_chr11_44780136_44780198, and Block_chr01_76299176_76299247) were found in at least three datasets (Supplementary Table S4b). The haploblock (block_chr05_4616945_4617251) and the QTN (chr05_7284066) detected on chromosome 5 were also identified by using the GAPIT-MLM GWAS model. By applying the second FDR<0.05 threshold, one significant QTN (chr05_11334011) and one haploblock (Chr05_4616945–4617251) were detected on chromosome 5 as highly significant (Supplementary Figure S3). Taken together, the marker Schr05_3842784 and the SNP Schr05_4617251, its haploblock loci (Block_chr05_4616945_4617251) were detected using two complementary statistical models as strongly associated with the maturity trait on chromosome 5.

#### 3.3.2 Drought

**FIGURE 7:**
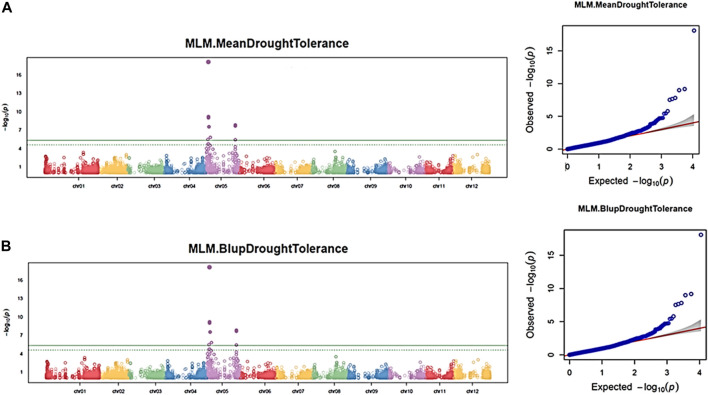
Manhattan and Q–Q plots showing QTN and chromosomal regions associated with the drought trait using the GAPIT–MLM. Each panel corresponds to one dataset. **(A)** Maturity mean dataset; **(B)** maturity BLUP dataset. The green line indicates the FDR threshold cut off <0.05, and the solid green line indicates the Bonferroni threshold cutoff at 0.05.

Similar to the maturity trait**,** by associating the 11,605 SNPs and the three independent drought phenotypic datasets, 17 significant SNPs (*p*-value <0.05) were associated with the drought trait in each dataset using the GAPIT–MLM. All significant SNPs were located on chromosome 5, and the Q–Q plot indicates a fairly well-fitted GWAS model (Figure 7). The mapped SNPs showed effects on the trait ranging from −0.56 to 0.48. Mann–Whitney non-parametric *U*-tests conducted on the 17 significant SNPs indicated that the markers Schr05_3842784 (*p* = 0.020), Schr05_5630468 (*p* = 0.048), and Schr05_7891044 (*p* = 0.036), which showed 7.4%, 7.24%, and 5.13% of PVE, respectively, had significant allelic effects, ranging from 0.26 to 0.32 in increasing the drought-tolerance index (Figure 6A). Noteworthy, the SNP Schr05_3842784 that showed a significant allelic effect (0.53) and 5.41% of PVE on maturity also showed a significant allele effect (0.32) and 7.4% of PVE on drought, suggesting a positive pleiotropic QTL effect on both traits (Figure 6). Again, the SNP Schr05_4617251, although not significant from the non-parametric *U*-test, showed 75% of PVE (Supplementary Table S4c).

A haploblock-based RTM-GWAS analysis was also conducted using the same SNP and drought phenotypic datasets. A total of five QTNs and three haploblock QTL loci were detected and found distributed on chromosomes 1, 2, 4, 5, 6, and 8. (Supplementary Table S4d). The two QTNs (chr06_11837397 and chr02_28184804) and three haploblock QTLs (chr05_4616945_4617251, chr05_4795074_4795187, and chr01_63508333_63508365) were detected in at last three datasets. By applying the second FDR<0.05 threshold, the haploblock Chr05_4616945_4617251 was detected on chromosome 5 as highly significant (Supplementary Figure S4).

Altogether, the GAPIT–MLM and RTM-GWAS models identified the SNPs Schr05_3842784, Schr05_5630468, Schr05_7891044, and Schr05_4617251, its haploblock QTL Chr05_4616945_4617251, respectively, as associated to drought, with Schr05_3842784 being pleiotropic for the maturity and drought traits with significant allelic affects (Supplementary Tables S4a–d).

### 3.4 Favorable alleles affecting the traits

The phenotypic differences between early (rated as 1) and late maturity (rated as 3) were notable, as were those between drought-susceptible (rated as 1) and -tolerant (rated as 2) clones. For maturity trait, Mann–Whitney non-parametric *U*-tests showed that 65% of accessions carried the AA genotype at Schr05_3842874 QTL, with an average maturity rate of 2.71, whereas 35% of accessions had the GG genotype, with an average maturity rate of 2.18.

**FIGURE 8:**
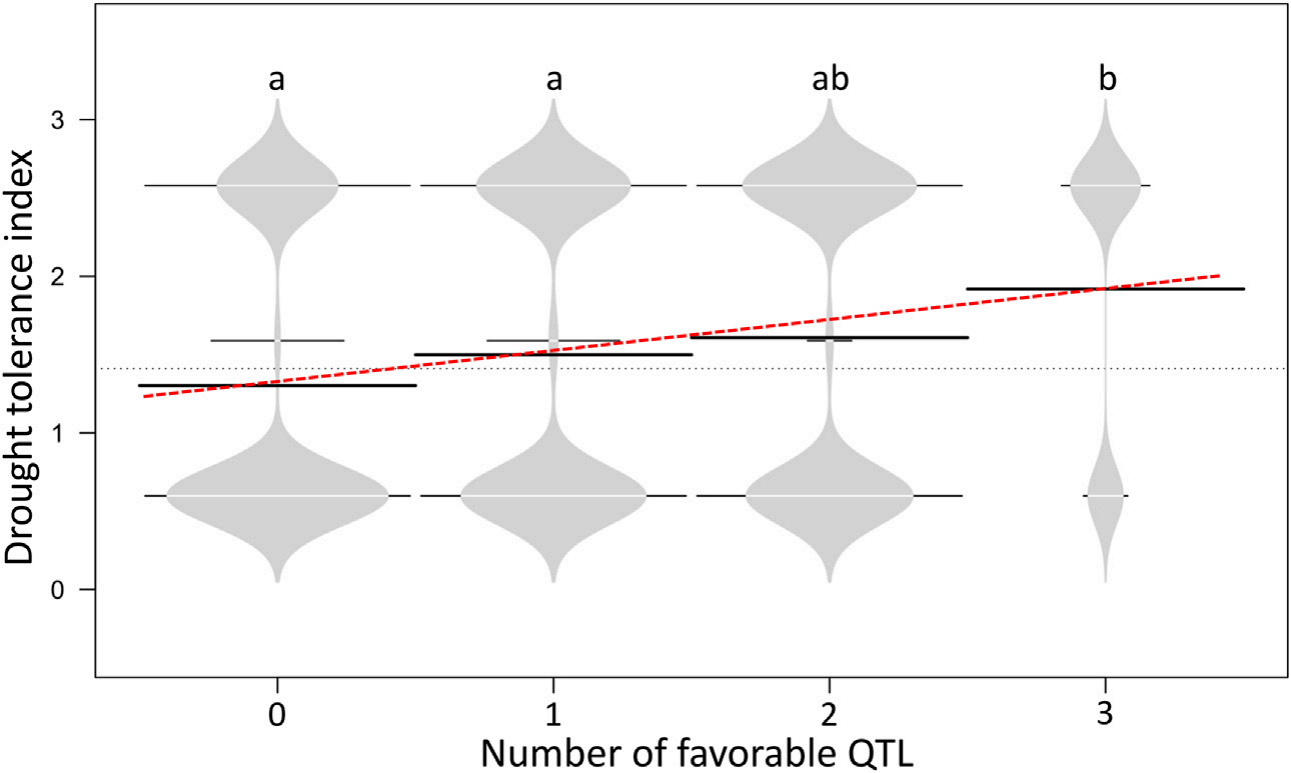
Bean plots illustrating the favorable QTL effect between potato genotypes harboring zero to three drought-tolerance QTLs. Horizontal black bars represent the mean for each class favorable QTL allele. Different letters indicate significant statistical differences according to the Kruskal–Wallis non-parametric test (*p* < 0.05).

In the current study, tolerance to drought was rated as 2 and susceptibility rated as 1. The G/A transition at the SNP site Schr05_3842874 was found to significantly impact the drought trait, and accessions carrying the AA genotype showed a drought-tolerance index of 1.5, while accessions harboring the alternative allele had a drought-tolerance index of 1.18. A positive linear trend was observed between the number of favorable QTLs and the drought-tolerance index (Figure 8), and no significant (*p* > 0.05) difference was observed between genotypes carrying 0 and 1 positive QTL, whereas accessions carrying 2 or 3 positive QTL alleles showed a statistically higher drought-tolerance index compared to those carrying 0 or 1 positive QTL (Figure 8).

### 3.5 Candidate genes associated with maturity and drought-tolerance traits

By scanning the 0.5 kb–10 kb genomic regions carrying of the 34 QTNs associated with the maturity trait, 38 candidate genes were detected. Similarly, 18 candidate genes were found associated with the drought trait (Supplementary Table S5). A total of 53% (20/38) and 50% (9/18) of the detected candidate genes were located within the 0.5–1 kb window screen surrounding the detected SNPs for maturity and drought traits, respectively. For the maturity trait, genes involved in auxin response (Soltu.DM.05G019140), anatomic structure development (Soltu.DM.06G019750), gibberellic acid signaling, leaf development, vegetative phase changes (Soltu.DM.05G012120), sucrose transport (Soltu.DM.05G006180), life span regulation (Soltu.DM.05G001990), transcription regulation (Soltu.DM.05G004540), and various cellular processes (Soltu.DM.05G020600, Soltu. DM.05G020610) were among others those found co-located with the 34 QTNs associated with maturity. Interestingly, the 0.5 kb−10 kb genomic region carrying the QTN Schr05_3842784 that showed favorable allele effects on the maturity trait harbored three candidate genes, including genes involved in apoptosis (Soltu.DM.05G004500), abscisic acid responses, and osmotic stress tolerance (Soltu.DM.05G004510) (Supplementary Table S5).

The 18 candidate genes co-located with QTNs associated with drought stress include genes involved in abscisic acid responses and osmotic stress tolerance (Soltu.DM.05G004510), response to endogenous stimulus (Soltu.DM.05G004530), transcription regulators for response to environmental stress and cues (Soltu.DM.05G004540), signal transduction and response to abiotic stress (Soltu.DM.05G005240 and Soltu. DM.05G008000), metabolic process and stress response (Soltu.DM.05G005260), chloroplast cellular component functions (Soltu.DM.05G005430 and Soltu. DM.05G008010), cellular and cell wall functions (Soltu.DM.05G006170, Soltu. DM.05G007510, Soltu. DM.05G020600, Soltu. DM.05G020590, and Soltu. DM.05G020610), and sucrose transport (Soltu.DM.05G006180) (Supplementary Table S5). The three QTNs with significant allele effects on the drought trait were associated with genes involved in sucrose transport (Soltu.DM.05G006180), gametogenesis, chloroplast and plant development (Soltu.DM.05G008010), signal transduction, and response to abiotic stress (Soltu.DM.05G008000) (Supplementary Table S5).

## 4 Discussion

Climate change is predicted to drastically impact crop productivity in the next decades (Dietz et al., 2021; Gervais et al., 2021; Hill et al., 2021; Nasir and Toth, 2022). Until recently, breeding for potato drought resistance has not been a priority objective in potato breeding, and only a limited number of drought-resistant potato varieties have been available (Sprenger et al., 2016; Martinez et al., 2021). By taking advantage of a mutagenized diploid potato collection we had previously developed (Somalraju et al., 2018), a germplasm panel of 384 clones was phenotyped for maturity and drought-tolerance traits in the field conditions, and GWAS were conducted. Here, a wide range of field maturity times, tolerance to drought, significant QTNs, and pleiotropic QTNs having favorable allelic effects on both maturity and drought traits and explaining 5.41% and 5.13%–7.4% of phenotypic variations, respectively, and candidate genes with potential known impacts on the two traits are reported. To our knowledge, this study is the first to report candidate genes associated with drought tolerance and/or maturity in narrow genomic regions of diploid potatoes.

Large genetic variations for maturity and drought traits were found in the population. Field testing for phenotypic traits is usually associated with GxE interactions (Allard and Bradshaw, 1964; Happ et al., 2021). In the current study, consistent maturity was observed over years for a large proportion (∼70%) of germplasm in the early- and late-maturing classes, while 30% in the moderate-to-late-maturity classes showed large variations. This observation suggests that early- and late-maturity phenotypes are less likely influenced by environmental factors but more genetically controlled. These two physiological states being life span strategies appear to be highly coordinately regulated by many senescence-associated genes (Lim et al., 2007; Guo et al., 2021), while the moderate-to-late-maturity clones may be more prone to both plant internal and external factors. Drought tolerance in crops has been extensively studied in controlled environments (Lahlou and Ledent, 2005; Schittenhelm et al., 2006; Aliche et al., 2018), with root length and volume, plant stem-type canopy, and leaf-type canopy characteristics being the most investigated traits following induced water shortage (Lahlou and Ledent, 2005; Schittenhelm et al., 2006; Aliche et al., 2018). In the current study, plant wilt was used as a drought-tolerance criterion under naturally weather-driven drought conditions in fields. Stable drought responses were observed for clones in the collection. Similar observations have been previously reported in rice (Ciu et al., 2018). Overall, nine clones rated as early to moderate maturing and tolerant to drought and 127 late-maturing and drought-tolerant potato clones were identified. These data suggest that some of the 136 clones can be climate-resilient to a short or long growing season while coping with drought stress.

Two advanced GWAS statistical models, the GAPIT–MLM and RTM-GWAS, were used in the current study to dissect the genetic architecture of maturity and drought-tolerance traits. In fact, it is well known that statistical models used in GWAS analyses can have a major impact on the output data (Yu et al., 2006; Tamba et al., 2017; Wen et al., 2018; Lo et al., 2019). Linkage QTL mapping and single-locus GWAS statistical models such as the GLM and MLM, GAPIT–MLM, and GAPIT–cMLM can detect large effect QTNs, whereas multi-locus models including mrMLM and RTM-GWAS increase the QTN detection power (Wang et al., 2016; Ren et al., 2018; Su et al., 2020; Wang and Zhang, 2021; Li et al., 2022a; 2022b; You et al., 2022). The MLMs are known for best fitting the kinship as a random effect that controls the genetic background caused by the genetic relatedness among individuals in the population. They theoretically correct the inflation from small polygenic effects, efficiently control the population stratification bias (Wen et al., 2018), reduce false positives (Liu et al., 2016), and generate QTLs as are in the linkage mapping. Hence, MLMs are good models for QTN/QTL detection. Nonetheless, the RTM-GWAS model exhibits the lowest false discovery rate as it imposes a very stringent correction criterion (He et al., 2017). Here, 34 and 17 QTNs were identified using the GAPIT–MLM for maturity and drought traits, respectively, whereas 30 and 8 QTNs/QTLs were detected by the RTM-GWAS model for the same two traits, respectively. Detection of fewer QTNs/QTLs by multi-locus GWAS models compared to single-locus GWAS models is common, as previously reported by Chen et al. (2019) when comparing the GAPIT–cMLM and mrMLM. By imposing the second-stage threshold of the RTM-GWAS model, only one and two QTN/QTLs were detected for the drought and maturity traits, respectively, thus confirming the stringency of the RTM-GWAS model. These models were complemented by the non-parametric *U*-test statistics for detecting favorable alleles potentially affecting the traits. For each trait, overlapping QTNs/QTLs were detected by using both models, and the two QTNs Schr05_3842784 and Schr05_4617251 were found to be pleiotropic for both maturity and drought traits. The finding suggests that these genomic regions strongly impact both traits and carry the genes that influence the traits. Indeed, along the non-parametric *U*-test-validated QTN Schr05_3842784, the QTN Schr05_4617251 located within the haploblock QTL chr05_4616945_4617251 was also found pleiotropic on maturity and drought, explaining 42% and 75% phenotypic variations, respectively. Nonetheless, chr05_4617251 was not statistically validated by the non-parametric *U*-test. The QTN Schr05_3842784, validated by all three models, showed 0.53 and 0.32 allele effects that increase the maturity and drought trait values and 5.41% and 7.4% of PVE, respectively. Furthermore, based on the three statistical models, three significant QTNs (Schr05_3842784, Schr05_5630468, and Schr05_7891044) were found associated to the drought trait, had 0.26–0.32 allelic effects that increase drought tolerance, and showed 5.13%–7.4% of PVE on the trait. The data reported here are significant because they derive from a unique and unprecedented mutagenized diploid germplasm panel. All previous mapping studies for the drought-tolerance trait have focused on bi-parental populations (Anithakumari et al., 2012; Khan et al., 2015; Schumacher et al., 2021); to our knowledge, no previous investigations have reported GWAS mapping for maturity and drought traits in diploid potatoes. Moreover, in the context of changing climate, the identification and development of early-maturity potatoes and potatoes that are drought tolerant are sought as a climate adaptation solution for short- and long-season growing areas. The current study identified both early- and late-maturing potato clones that are drought-tolerant, meaning that maturity class can be a physiological variable that can be used to mediate drought tolerance in crops through drought escape (Hill et al., 2021).

In the current study, a total of 38 candidate genes were identified in genomic regions harboring the 34 QTNs associated with maturity, while 18 candidate genes were found for the 17 QTNs associated with the drought trait. Most of the genes were identified in a close vicinity (0.5–1 kb) of the QTNs, suggesting their true impact on the traits. In their study using an indirect yield trait (DRYM) for drought tolerance in tetraploid potatoes, Schumacher et al. (2021) reported candidate genes for drought tolerance, albeit from very large (1.3 Mb–36.76 Mb) genomic regions. Our data are, thus, highly relevant for targeted gene studies and marker development when compared with the report by Schumacher et al. (2021). The roles of auxin, gibberellic acid, and sucrose transport in plant development are well known (Nagar et al., 2021). Furthermore, the roles for phytohormones abscisic acid, brassinosteroid, cytokinin, ethylene, gibberellic acid, jasmonic acid, and salicylic acid have been reported to be crucial in drought stress tolerance in plants through the regulation of cellular functions and signaling (Guo et al., 2021; El Sabagh et al., 2022). Our data showed genes such as the *auxin response factor* that mediates auxin response and plays a crucial role in lateral root development (El Sabagh et al., 2022); *squamosa*, which is involved in gibberellic acid signaling, leaf development, and vegetative phase change; the *longevity assurance gene (LAG1)*, which controls the life span; and *sucrose transporter* as part of the genes found to carry the QTNs associated to the maturity trait. Importantly, the QTN Schr05_3842784 with a validated allelic effect on maturity was associated with genes involved in ubiquitination, apoptosis, cell death (Soltu.DM.05G004500) (Shen et al., 2018), abscisic acid responses, and osmotic stress tolerance (Soltu.DM.05G004510). These observations suggest that critical responsive genes carrying SNP variants associated with the maturity trait were efficiently mapped in the current study. Similarly, water access and conservation in the plant tissue, chloroplast stability, and its proper development are critical for plant survival under stress (He et al., 1995; Anithakumari et al., 2012; Gervais et al., 2021; Hill et al., 2021; Lin et al., 2021). Here, using a visual wilting assessment of the drought trait in the field condition and followed by a GWAS, candidate genes playing roles in abscisic acid responses and osmotic stress tolerance, responsive genes to an endogenous stimulus, transcription regulators for response to environmental stress and cues, signal transduction and response to abiotic stress (Soltu.DM.05G008000), various stress-responsive genes, chloroplast and cellular component function gene (Soltu.DM.05G005430), cellular process and cell wall function genes (Soltu.DM.05G004500 and Soltu. DM.05G020590), and sucrose transport (Soltu.DM.05G006180) were identified as associated with drought stress. The role for abscisic acid as a signaling regulator of stomatal conductance and leaf thickness has been reported as drought-tolerance mechanisms (Wang et al., 2010; Hill et al., 2021). Our data show genes involved in signal transduction and response to abiotic stress (Soltu.DM.05G005240, Soltu. DM.05G005260, and Soltu. DM.05G008000), abscisic acid signaling (Soltu.DM.05G004510), chloroplast development (Soltu.DM.05G008010) (Lin et al., 2021), ands cell wall modifications (Soltu.DM.05G020590), all concurring in synergy to drought tolerance. Although similar genes have been reported in other crops under drought stress (Mathew et al., 2019; Soto-Cerda et al., 2020; Gutierrez et al., 2023), no such close intragenic QTNs associated with drought tolerance have been previously reported in potato. Our findings strongly suggest that relevant SNP markers associated with drought-tolerance genes can be developed from the current study. Based on the existing knowledge, cultivated tetraploid potato landraces belonging to *S. tuberosum*, group Andigena, carry some drought-tolerance traits (Nasir and Toth, 2022), and the current data are contributory to expanding potato germplasm with drought tolerance while displaying diverse maturity classes for diverse continental agrosystems.

In conclusion, this study deciphered the genetic architecture as it relates to QTN/QTL associated with maturity and drought traits in a diploid germplasm panel and provided the first evidence for the presence of candidate genes conferring tolerance to maturity and drought stress on chromosome 5 of diploid potatoes. The genetic loci with high phenotypic effects on both maturity and drought traits can be of worth interest for marker development and further detailed functional studies. The data and germplasm herein reported are prime genetic resources for breeders and biologists in conventional breeding and targeted gene editing in the context of climate change.

## Data Availability

The original contributions presented in the study have been deposited in NCBI public database under SRA accession PRJNA1032882.

## Supplementary material

The Supplementary Material for this article can be found online at: https://www.frontiersin.org/articles/10.3389/fgene.2023.1306519/full#supplementary-material

Supplementary Figure S1 Temperature (°C) and precipitation (mm) data during growing seasons in years 2020 to 2022.

Supplementary Figure S2 Density plot of genome-wide distribution of 11,605 SNP markers across the diploid potato genome. The horizontal axis displays the chromosome length (Mb), while the colors represent SNP density.

Supplementary Figure S3 Manhattan plot showing QTN/QTL and chromosomal regions associated with maturity trait using the RTM-GS model. The blue line indicates the second FDR threshold cut off <0.05.

Supplementary Figure S4 Manhattan plot showing QTN/QTL and chromosomal regions associated with drought trait using the RTM-GWAS model. The blue line indicates the second FDR threshold cut off <0.05.
