# Supplementary figures and images for: Genome-wide genetic architecture for plant maturity and drought tolerance in diploid potatoes
Source: Front Genet. 2024 Jan 31;14:1306519. doi: 10.3389/fgene.2023.1306519 (PMC10864671; doi:10.3389/fgene.2023.1306519)

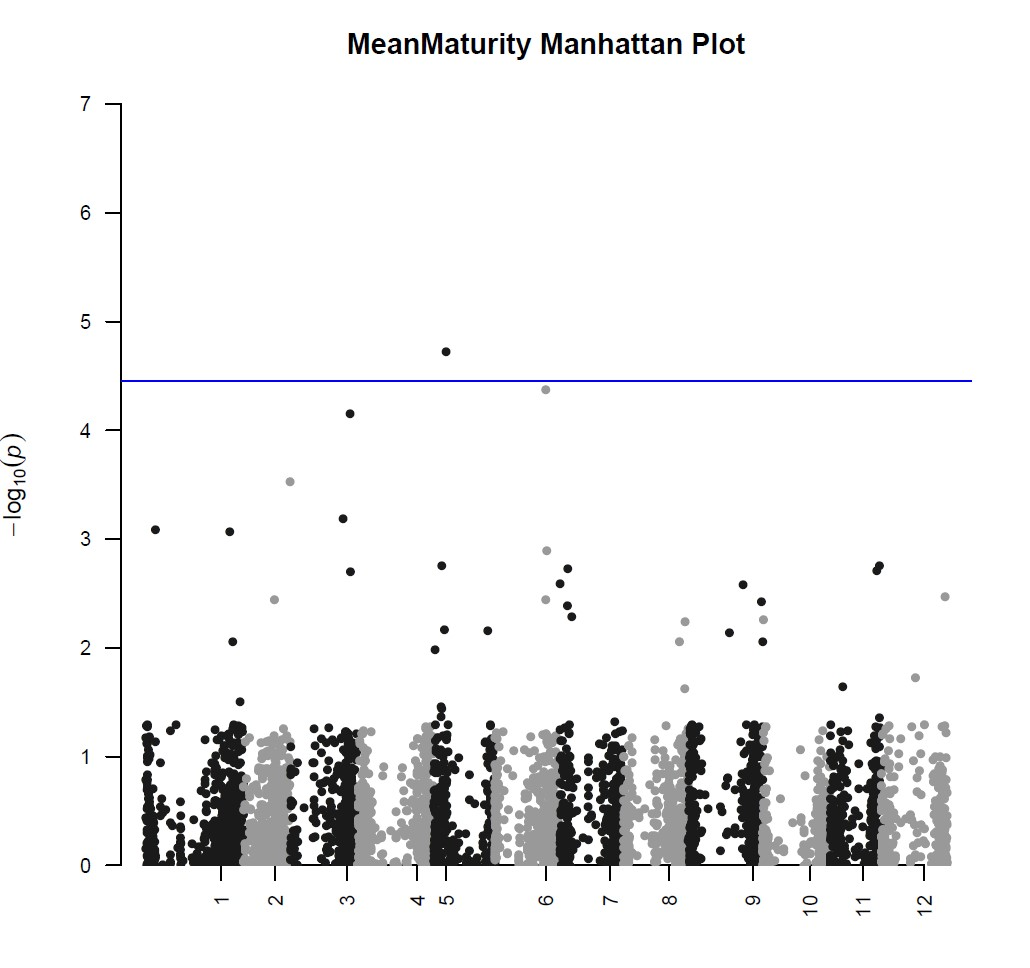

Supplement: Supplementary file 1 [file Image3.TIFF]

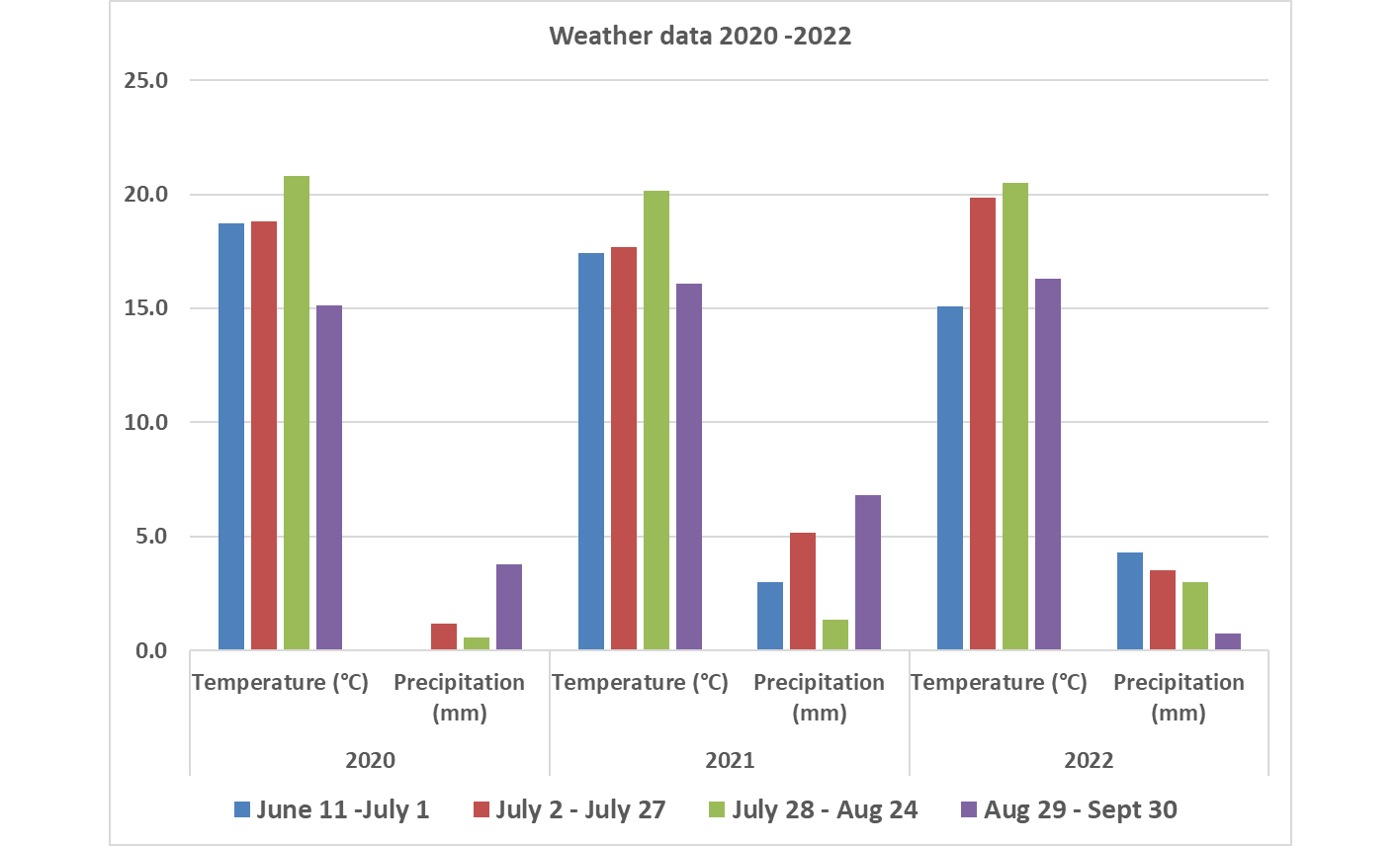

Supplement: Supplementary file 2 [file Image1.TIFF]

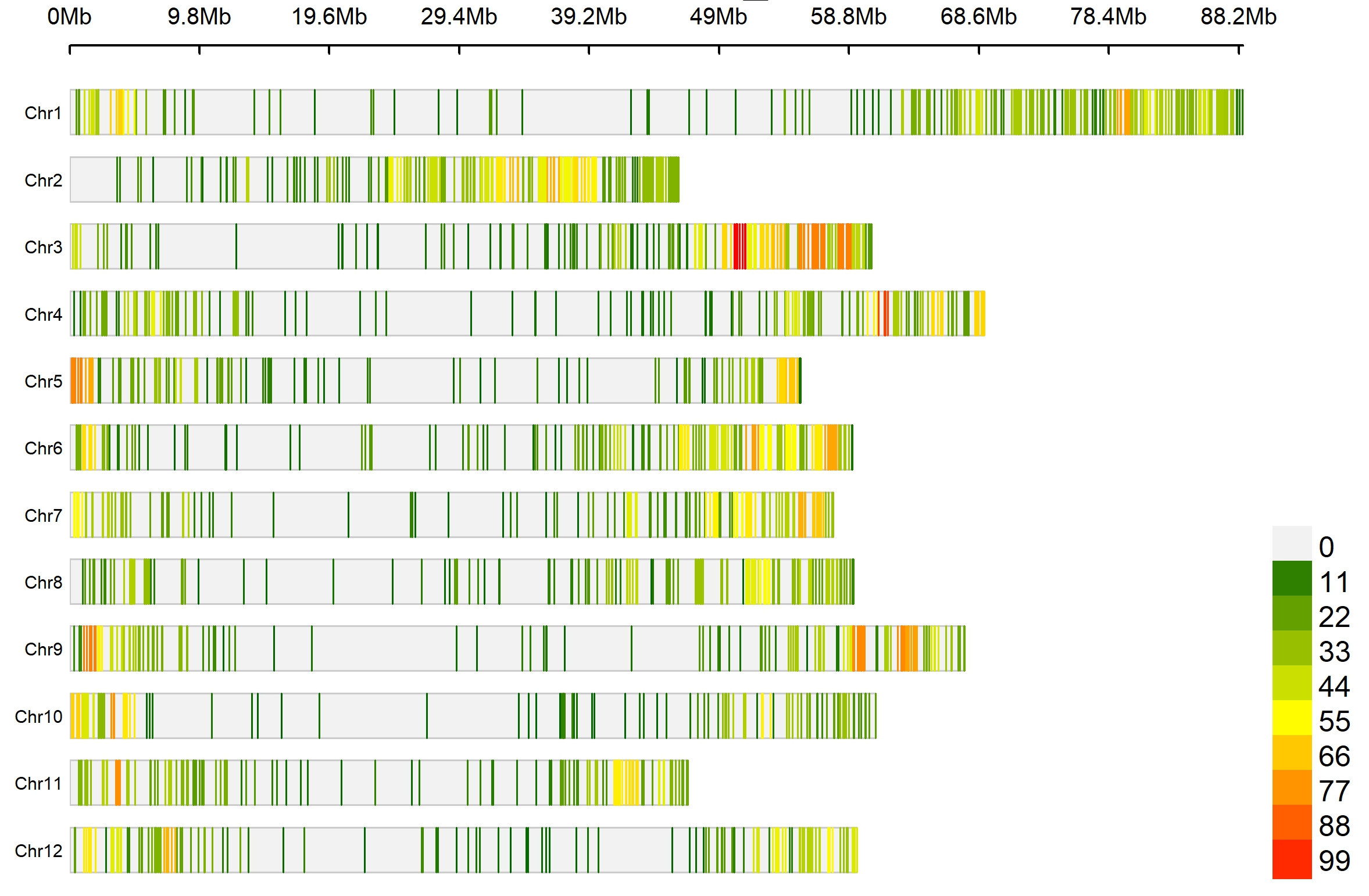

Supplement: Supplementary file 8 [file Image2.TIFF]

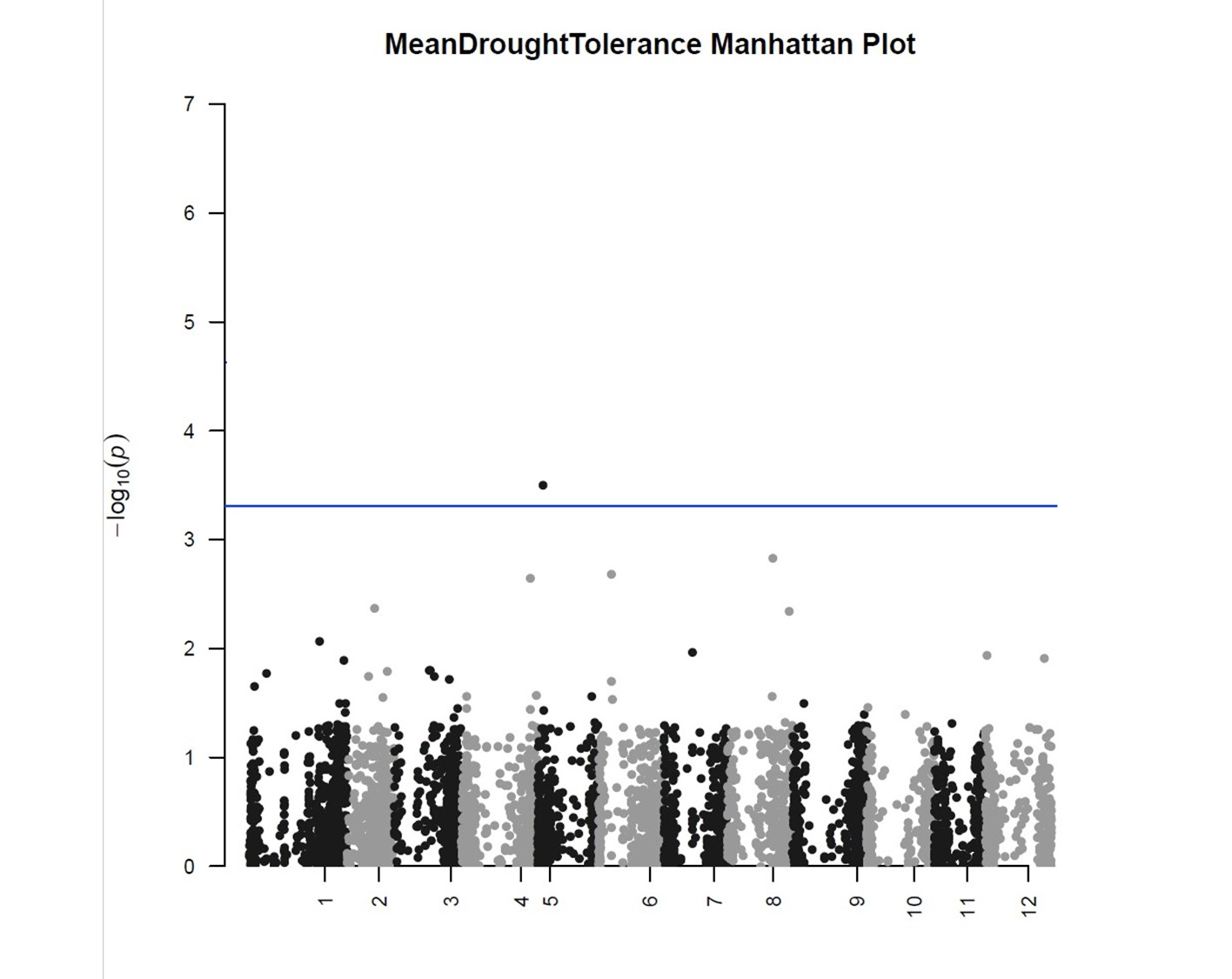

Supplement: Supplementary file 9 [file Image4.TIFF]
