# Supplementary material for: Genome-wide genetic architecture for plant maturity and drought tolerance in diploid potatoes
Source: Front Genet. 2024 Jan 31;14:1306519. doi: 10.3389/fgene.2023.1306519 (PMC10864671; doi:10.3389/fgene.2023.1306519)
Supplement: Supplementary file 4 [file Table2.DOCX]

**Supplementary Table 2**. Episodes of drought with corresponding temperature (C) and precipitation (mm) data during growing seasons in years 2020 and 2022. Episodes after which drought data were collected are shown for each year.

|  | **2020** | | | **2022** | | |
| --- | --- | --- | --- | --- | --- | --- |
| Drought periods | Duration (Day) | Temperature (°C) | Precipitation (mm) | Duration (day) | Temperature (°C) | Precipitation (mm) |
| June 11 - Jul 1 | 21 | 18.20 | 0.00 | . | . | . |
| July 12 - July 27 | 16 | 19.60 | 0.23 | . | . | . |
| July 20 - July 28 | . | . | . | 8 | 22.30 | 0.04 |
| July 31 - Aug 7 | . | . | . | 8 | 22.05 | 0.41 |
| July 31 - Aug 24 | 25 | 20.64 | 0.07 | . | . | . |
| Aug 28 - Sept 15 | . | . | . | 19 | 17.80 | 0.24 |
