# Supplementary material for: Genome-wide genetic architecture for plant maturity and drought tolerance in diploid potatoes
Source: Front Genet. 2024 Jan 31;14:1306519. doi: 10.3389/fgene.2023.1306519 (PMC10864671; doi:10.3389/fgene.2023.1306519)
Supplement: Supplementary file 6 [file Table3.DOCX]

**Supplementary Table 3**. Restricted maximum likelihood analysis (REML) of maturity and drought traits.

| Source | d.f. | Maturity  Mean square | Wald statistic | Drought_tolerance  Mean square | Wald statistic |
| --- | --- | --- | --- | --- | --- |
| Stratum | - | - |  | - | - |
| Clone | 382 | 1.024 | <0.001 | 0.476 | <0.001 |
| Year | 1 | 6.039 | <0.001 | 0.03255 | 0.059 |
| Residual | 382 | 0.2093 |  | 0.009053 |  |
| Total | 765 | - |  | - | - |
| Mean |  | 2.337 |  | 1.41 |  |
| S. error |  | 0.0233 |  | 0.005 |  |
| Range |  | 2.304 – 2.369 |  | 1.403 – 1.417 |  |
